# Room Size Influences Flow in Robotic-Assisted Surgery

**DOI:** 10.3390/ijerph18157984

**Published:** 2021-07-28

**Authors:** Falisha Kanji, Tara Cohen, Myrtede Alfred, Ashley Caron, Samuel Lawton, Stephen Savage, Daniel Shouhed, Jennifer T. Anger, Ken Catchpole

**Affiliations:** 1 Department of Surgery, Cedars-Sinai Medical Center, Los Angeles, CA 90048, USA; tara.cohen@cshs.org (T.C.); caronash@msu.edu (A.C.); daniel.shouhed@cshs.org (D.S.); jennifer.anger@cshs.org (J.T.A.); 2 Department of Anesthesia and Perioperative Medicine, Medical University of South Carolina, Charleston, SC 29425, USA; alfredm@musc.edu (M.A.); lawtons@musc.edu (S.L.); catchpol@musc.edu (K.C.); 3 Department of Surgery, Medical University of South Carolina, Charleston, SC 29425, USA; savages@musc.edu

**Keywords:** operating room, robotic-assisted surgery, layout, operating room design, flow disruptions

## Abstract

The introduction of surgical technology into existing operating rooms (ORs) can place novel demands on staff and infrastructure. Despite the substantial physical size of the devices in robotic-assisted surgery (RAS), the workspace implications are rarely considered. This study aimed to explore the impact of OR size on the environmental causes of surgical flow disruptions (FDs) occurring during RAS. Fifty-six RAS procedures were observed at two academic hospitals between July 2019 and January 2021 across general, urologic, and gynecologic surgical specialties. A multiple regression analysis demonstrated significant effects of room size in the pre-docking phase (t = 2.170, df = 54, β = 0.017, *p* = 0.035) where the rate of FDs increased as room size increased, and docking phase (t = −2.488, df = 54, β = −0.017, *p* = 0.016) where the rate of FDs increased as room size decreased. Significant effects of site (pre-docking phase: *p* = 0.000 and docking phase: *p* = 0.000) were also demonstrated. Findings from this study demonstrate hitherto unrecognized spatial challenges involved with introducing surgical robots into the operating domain. While new technology may provide benefits towards patient safety, it is important to consider the needs of the technology prior to integration.

## 1. Introduction

Surgical procedures are complex tasks that not only place a great degree of responsibility on the operating room (OR) staff but also create substantial demands on the environment in which they are performed. Although the architectural design of ORs varies across hospitals, one commonality is that most ORs were built when surgical technology was not as advanced as it is today [1]. Current surgical technologies, such as those used in robotic-assisted surgery (RAS), often demand more space and require the installation or inclusion of additional equipment, such as monitors, consoles, and a tower, for it to be successful. Furthermore, the introduction of such advanced surgical technology requires accommodation for additional sterile fields, changes to room setup, and to the overall movement and flow of both equipment and personnel [2,3,4]. Accommodations are made within the OR for the introduction of advanced surgical technology. However, it is important to note that these accommodations can change when impacted by other factors such as global health crises (e.g., COVID-19 and complete cessation of elective cases), further impacting the general flow of RAS [5]. The general challenges with creating accommodations for such technology can be attributed to a number of factors including, but not limited to, OR size and limitations with respect to workspace design [6,7,8]. Additionally, the challenges associated with accommodating advanced surgical technology can affect a variety of factors such as the flow of tasks, patient safety [6,7], OR staff safety, storage, location of instruments, sterile protocols, and teamwork [9]. Therefore, there is a need to evaluate OR size as it relates to high-technology integration.

One approach to studying high-technology integration into the surgical environment involves observing and documenting flow disruptions (FDs) or “*deviations from the natural progression of an operation, thereby potentially compromising the safety of the operation*” [10] (p. 660). Through the observation and documentation of FDs, it has been possible to understand better the contributing factors that lead to challenges with teamwork, task design, and patient safety as it relates to RAS [11,12]. Observations can include evaluating the workflow of the circulating nurse, surgical technician, and other OR staff through different phases of RAS and evaluating the OR layout prior to robot docking, during robot docking, when the surgeon is on the console, and after robot undocking (i.e., movement and placement of trash cans, stools, tables, trays, monitors, etc.). These evaluations can provide insight into the challenges faced when using surgical robots in ORs that are not designed for such technology.

While several studies have explored the role of FDs in RAS, to our knowledge, none have investigated the role of OR size on the RAS work-system. Therefore, the focus of our study was to investigate workspace related FDs to better understand the impact of the physical environment on safety and efficiency in RAS.

## 2. Materials and Methods

This prospective direct observational study was conducted between July 2019 and January 2021 (18 months) at two hospital sites. Site 1: is an 886-bed non-profit academic hospital in Southern California and site 2 is a 700-bed non-profit academic medical center in South Carolina. All research activities were approved by the Institutional Review Board at both study sites (site 1: Pro0005624; site 2: Pro00088741).

Prior to data collection, researchers (*n* = 4 across the two institutions) were trained to observe RAS procedures and FDs in the OR. Training included a review of literature and practiced observations in the OR. Literature included an introduction to the overall larger study for the purpose of understanding the project’s aims and methodology and published literature of studies investigating FDs in RAS. Observers conducted five practice observations with an experienced observer to acclimate to the OR and learn to use the data collection tool prior to independent data collection.

**Figure 1:**
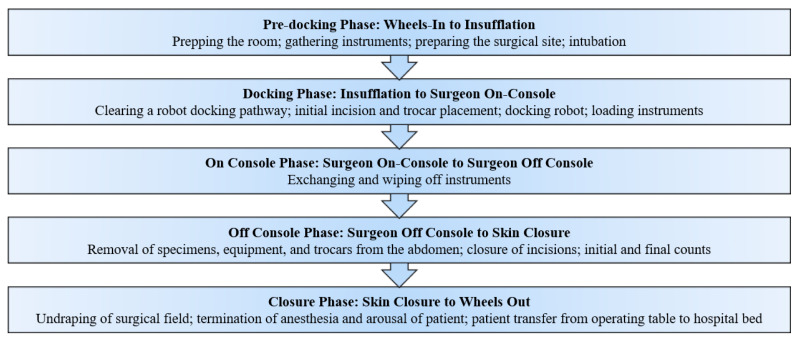
Tasks conducted by the OR team during RAS phases.

RAS procedures conducted using the da Vinci Si or Xi robot in the following surgical specialties were selected for observation: (1) general surgery (hernia repair; cholecystectomy; and gastrectomy); (2) urology/urogynecology (simple and radical prostatectomy; nephrectomy; sacrocolpopexy); and (3) gynecology (hysterectomy for benign and malignant conditions; excision of endometriosis). Each procedure was broken into five phases that corresponded with major transitions in RAS (see Figure 1).

Data was collected by trained observers over the course of 18 months. While in the OR, observers used a data collection tool developed by the research team using Microsoft Excel, to document FDs that occurred in each of the five phases [3]. FDs were timestamped and recorded in the form of a written narrative and were then categorized into one of ten previously described major categories (communication, coordination, environment, equipment, external factors, other, patient factors, surgical task considerations, training, unsure) [11]. For this study, the focus was on FDs related to the environment of the OR (i.e., FDs relating to issues within the physical environment), thus, those FDs categorized as “environment” were extracted and evaluated. Following data collection, one researcher subclassified the environmental FDs into minor categories based on the narrative provided. This included observed deviations related to wires and cords, lighting, temperature, narrow or tight spaces, architectural design, and placement and movement of equipment (e.g., trashcans, chairs, trays, etc.). Square footage and floor plans for each OR where RAS cases were observed were also obtained for analysis.

IBM SPSS (version 24) statistical software (IBM Corp. Released 2016. IBM SPSS Statistics for Windows, Version 24.0. Armonk, NY, USA: IBM Corp.) was utilized to conduct statistical analyses for this study. Multiple regression analysis was conducted to determine the effect of room size and hospital site on the rate of environmental FDs (average number of FDs occurring per hour) by and across all five phases of surgery. Significance was assessed at the alpha = 0.05 level.

## 3. Results

**Figure 2:**
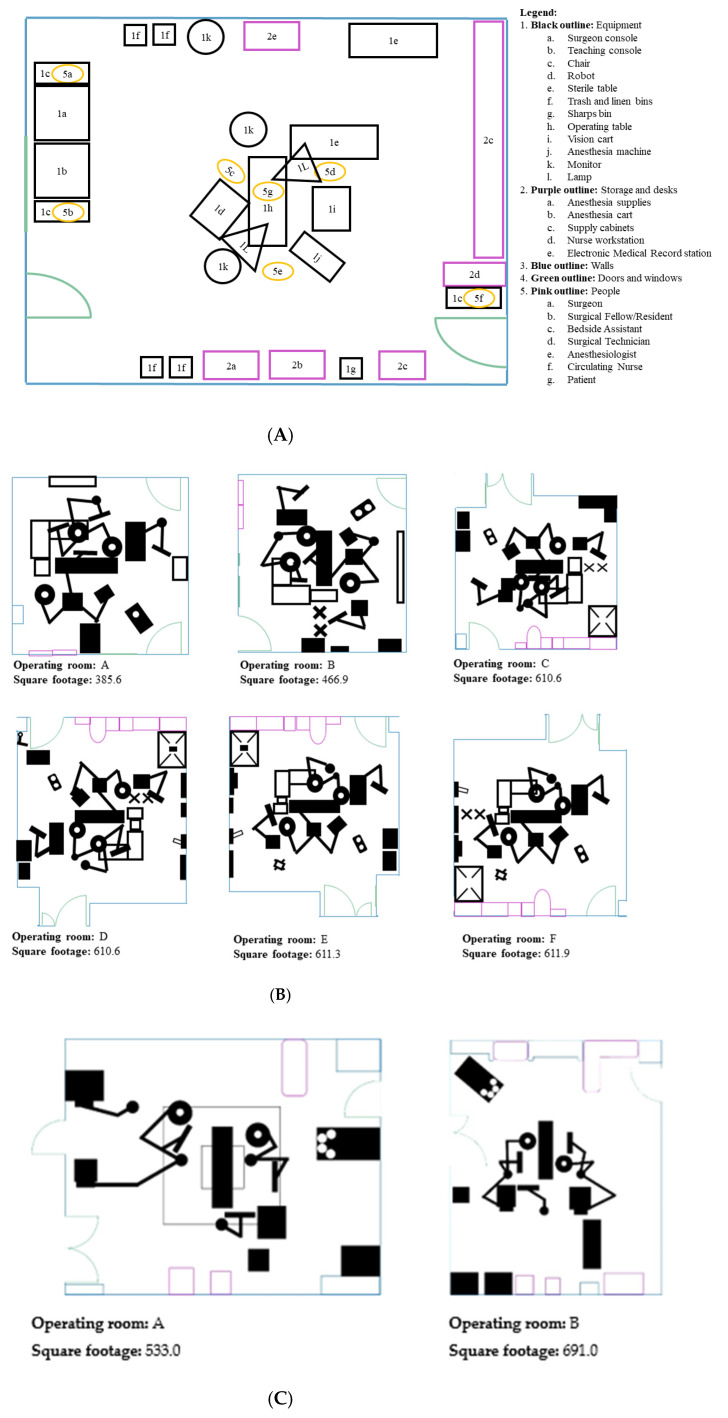
(**A**) A mock-up of an RAS OR during the on-console phase to demonstrate a general layout of a robotic operating room, (**B**) operating room floor plans for site 1, (**C**) operating room floor plans for site 2.

A total of 56 observations were conducted in eight different ORs across the two sites (site 1: *n* = 28, site 2: *n* = 28). OR sizes varied from 385.6 square feet to 691.0 square feet (see Table 1 and Figure 2A–C). Of the 670 environmental FDs that were observed during the 56 observations, 491 environmental FDs (*n* = 129 (26.27%) in the pre-docking phase and n = 110 (22.40%) in the docking phase) were observed at site 1 and 179 environmental FDs (*n* = 54 (30.17%) in the pre-docking phase and *n* = 25 (13.97%) in the docking phase) were observed at site 2.

**Figure 3:**
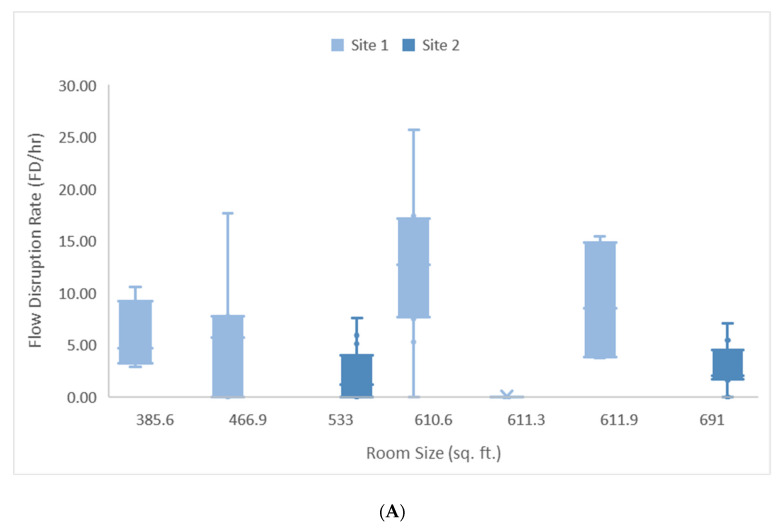

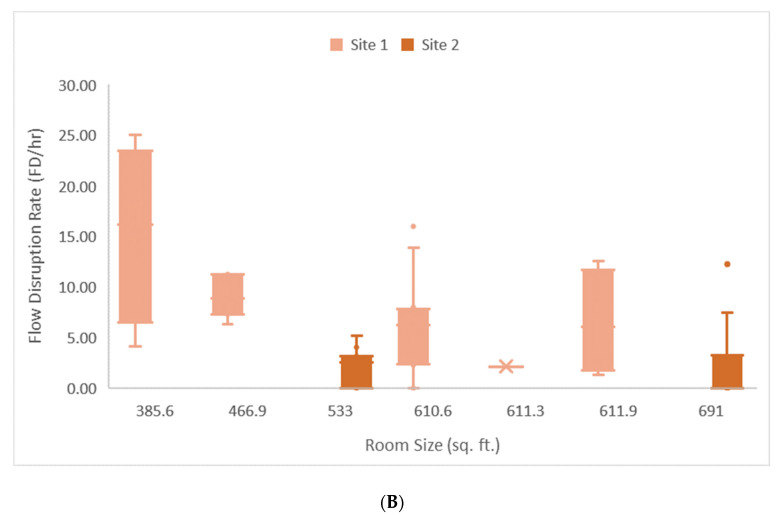
(**A**) Rate of environmental flow disruptions during the pre-docking phase for sites 1 and 2. (**B**) Rate of environmental flow disruptions during the docking phase for sites 1 and 2.

Pre-docking Phase: Room size (t = 2.170, df = 54, β = 0.017, *p* = 0.035) and site (t = −5.423, df = 54, *p* = 0.000) had a significant effect on the rate of environmental FDs occurring during phase 1 (R2 = 0.357). For every 100 square foot increase in room size, the rate of environmental FDs increased by 1.7 per hour, with site 1 experiencing 7.5 more FDs per hour than site 2 (see Figure 3A).

Docking Phase: Room size (t = −2.488, df = 54, β = −0.017, *p* = 0.016) and site (t = −3.908, df = 54, *p* = 0.000) had a significant effect on the rate of environmental FDs occurring during phase 2 (R2 = 0.380). In contrast to phase 1, for every 100 square foot decrease in room size the rate of environmental FDs increased by 1.7 per hour, with site 1 experiencing 4.9 more FDs per hour from a baseline of 17.5 FDs per hour (see Figure 3B).

The multiple regression did not demonstrate significant effects of room size on environmental FDs for phase 3, 4, 5, and across all five phases.

**Figure 4:**
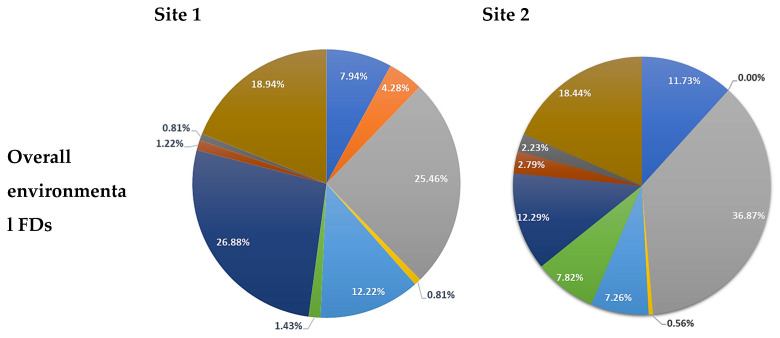

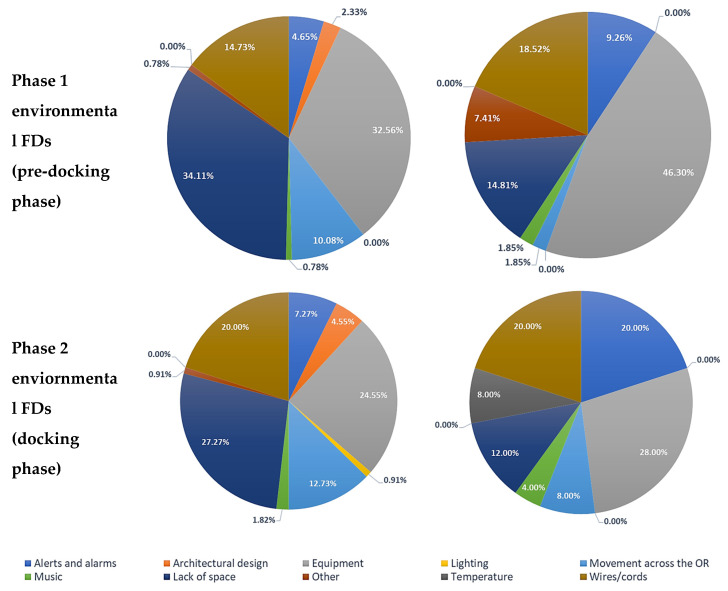
Percentage of environmental FDs at site 1 and site 2.

Across sites, the highest rate of environmental FDs were caused by equipment (site 1: 25.46% and site 2: 36.87%), wires and cords (site 1: 18.94% and site 2: 18.44%), and lack of space (site 1: 26.88% and site 2: 12.29%). With respect to phases, the majority of environmental FDs during the time period between wheels-in to insufflation (pre-docking phase) and insufflation to robot docking (docking phase) at site 1 were caused by limited space (pre-docking phase: 34.11% and docking phase: 27.27%) compared to site 2 where the cause was due to equipment (pre-docking phase: 46.30% and docking phase: 28.00%) (see Figure 4).

## 4. Discussion

Our study explored the relationship between OR size and environmental FDs active in RAS. Observations were conducted during general, urologic, and gynecologic RAS over an 18-month period. A significant effect of room size and site on the rate of environmental FDs were found for the pre-docking and docking phases. With respect to the pre-docking phase, as room size increased, so did the rate of environmental FDs. Conversely, during the docking phase, as room size decreased, the rate of environmental FDs increased. However, a significant effect of room size was not found for the on console, off console, and closure phases. This may be because, unlike the pre-docking and docking phases, tasks are not completed in parallel (i.e., OR staff rarely cross paths while conducting tasks) and there are fewer tasks in general that are conducted in these phases.

Observations were conducted in a range of OR sizes from 385.6 sq. ft. to 691 sq. ft. In the larger ORs, OR size had an effect on environmental FDs where issues consisted of problems with wires and cords, equipment causing unnecessary congestion, and increased movement around the OR. More specifically, these issues were found during the pre-docking phase as the OR staff prepared the room and the patient for the RAS procedure. During this period, preparation of the room includes draping the robotic arms, organizing sterile instruments on surgical trays and sterile tables, retrieving necessary equipment or removing equipment that is no longer needed, and retrieving any remaining supplies from closets and cabinets located within or outside the OR. Although these preparatory tasks are a normal part of the process, they were negatively impacted by the size of the OR.

Alternatively, in smaller ORs, the size of the OR has a different effect on the type of FDs that occurs. More specifically, the observations revealed that OR teams face more issues in smaller ORs during the time between insufflation to when the robot is docked at the operating table during the docking phase because of the increased movement of equipment around the room. When insufflation begins, the circulating nurse and other available OR staff prepare for robot docking by rearranging equipment around the room to clear a pathway for the robot (i.e., moving trash cans, overhead lights, monitors, etc.) [11] and ensure that all supplies needed for the robotic procedure are available within the OR. The rearrangement of equipment and machines around the OR requires a specific amount of space to aid in avoiding collision of equipment and creation of congestion as equipment and machines are repositioned around the OR. Unlike the preceding phase, equipment rearrangement and robot docking are less problematic in larger ORs than they are in smaller ORs because of the increased size of the OR.

Although the activities between the two phases are similar such that both involve preparation and increased movement around the room, OR size contributes differently to environmental FDs. When assessing differences across sites, the rate of environmental FDs was higher at site 1 than at site 2 for both the pre-docking and docking phases. Factors that could have contributed to differences in the rate of environmental FDs could be attributed towards differences in the dynamics between OR teams across sites and its effect on the observers, or vice versa [13], differences in the way organizations conduct RAS training for OR team members, differences across organizations with respect to the way OR teams prepare for RAS procedures (e.g., briefings, surgeons arriving to the OR before case starts to assist with preparation, etc.), and OR setup specifically with regards to supplies being readily available and equipment placement.

This study identified some of the challenges associated with integrating advanced technology into a surgical environment not originally designed for its accommodation. We found that impact of the size of the room varied based on the phase of surgery: OR room setup (pre-docking phase) could be performed more efficiently if the ORs were smaller, while docking of the robot (docking phase) could be performed best when ORs were larger. Because RAS procedures involve more than just the OR staff, this information can help inform both internal and external teams how materials and supplies might be organized in the room during setup and at different phases of surgery [14]. The improved organization of the room could decrease unnecessary movement while reducing the number of FDs [8]. Additionally, it may be beneficial to provide pathways or diagrams for efficient movement during the second phase of surgery (docking phase), especially in smaller rooms. Furthermore, with respect to design, this information can help healthcare architects plan the design and organization of OR suites that will be utilized for RAS [12,15,16]. For example, it may be possible to develop innovative rooms that allow for reachable equipment and wires to accommodate for tasks occurring during the pre-docking phase, but larger ORs to accommodate the movement of equipment and positioning of the robot [17] during the docking phase. Thus, the successful implementation of RAS benefits from both careful considerations about the size of the OR in which the surgeries are to be conducted; and on a team who recognizes the importance of organizing the OR to account for the demands of the surgery within the physical limitations of the OR.

### Limitations

While this study focused on environmental FDs because they were more likely to be affected by room size, it is possible that room size contributes to other issues in the broader system context including communication, coordination, teamwork, and OR staff training. However, our study was not configured to specifically identify room-related issues within these wide observational categories, nor was it configured to estimate an ideal OR size for RAS procedures. Furthermore, this study did not investigate the impact of environmental FDs on safety and the differences in OR layout, more specifically room size and shape, and its effect on workflow in RAS. Future research should be conducted to investigate ideal OR size for RAS procedures and identify the influence of non-environmental FDs and their impact on room-related issues. Although our team at both sites trained together and had high-inter-rater reliability, it is also possible that there was variation in observation techniques, leading one site to measure more flow disruptions than the other. It is also possible that other unmeasured variables contributed to our results. For example, if better trained robotic staff were more likely to work in the smaller ORs, we may have underestimated flow disruptions caused by room size. Future work should aim to conduct equal numbers of observations in each room size, all with the same staff and same types of procedures, to decrease variability within the data.

## 5. Conclusions

Although new technology provides greater benefits towards the surgical process for both the surgeon and the patient, it is important to consider the efficient use of the technology taking OR size into consideration before it is integrated into the OR for use. The results from this study found that environmental FDs varied based on tasks and phases in which those tasks were conducted. Therefore, appropriate accommodations, such as scheduling RAS procedures in ORs that are large enough to accommodate both the robotic equipment and workflow, must be made to successfully integrate new technology within the OR to provide greater efficiency across tasks and phases in RAS. Given the broader systems implications and requirements for successful RAS compared to less equipment-heavy operations, optimal room sizes can be found for high-technology surgeries.

## Figures and Tables

**Table 1:** Overview of room size and RAS procedures observed during this study.

Site	Room	Square Footage	*N*	% of Procedures by Site	RAS Procedures Observed
1	A	385.6	4	14.29%	Hysterectomy, hernia repair, prostatectomy, sacrocolpopexy,
	B	466.9	7	25.00%	cystoscopy, hysterectomy, sacrocolpopexy, salpingectomy, prostatectomy
	C	610.6	3	10.71%	Excision of endometriosis, fundoplication, hernia, sleeve gastrectomy
	D	610.6	9	32.14%	Hernia repair, sleeve gastrectomy
	E	611.3	1	3.57%	Hernia repair
	F	611.9	4	14.29%	Cholecystectomy, cystoscopy, fundoplication, hysterectomy, hernia Repair, salpingectomy
2	A	533	15	53.57%	Hysterectomy, nephrectomy, prostatectomy, sacrocolpopexy, hernia repair
	B	691	13	46.43%	Hernia repair, hysterectomy, sacrocolpopexy

## Data Availability

Not applicable.

## References

[1] Talamini M.A., Chapman S., Horgan S., Melvin W.S. (2003). A prospective analysis of 211 robotic-assisted surgical procedures. Surg. Endosc. Other Interv. Tech..

[2] Berman J., Dajer E., Fong Y. (2018). Robotic operating rooms. The Sages Atlas of Robotic Surgery.

[3] Catchpole K., Perkins C., Bresee C., Solnik M.J., Sherman B., Fritch J., Gross B., Jagannathan S., Hakami-Majd N., Avenido R. (2016). Safety, efficiency and learning curves in robotic surgery: A human factors analysis. Surg. Endosc..

[4] Souders C.P., Catchpole K.R., Wood L.N., Solnik J.M., Avenido R.M., Strauss P.L., Eilber K.S., Anger J.T. (2017). Reducing operating room turnover time for robotic surgery using a motor racing pit stop model. World J. Surg..

[5] Khaleghi A., Mohammadi M.R., Jahromi G.P., Zarafshan H. (2020). New ways to manage pandemics: Using technologies in the era of covid-19: A narrative review. Iran. J. Psychiatry.

[6] Taaffe K., Joseph A., Khoshkenar A., Machry H., Allison D., Reeves S.T. (2020). Proactive evaluation of an operating room prototype: A simulation-based modeling approach. J. Patient Saf..

[7] Wahr J.A., Prager R.L., Abernathy Iii J.H., Martinez E.A., Salas E., Seifert P.C., Groom R.C., Spiess B.D., Searles B.E., Sundt Iii T.M. (2013). Patient safety in the cardiac operating room: Human factors and teamwork: A scientific statement from the american heart association. Circulation.

[8] Ahmad N., Hussein A.A., Cavuoto L., Sharif M., Allers J.C., Hinata N., Ahmad B., Kozlowski J.G., Hashmi Z., Bisantz A. (2016). Ambulatory movements, team dynamics and interactions during robot-assisted surgery. BJU Int..

[9] Jain M., Fry B.T., Hess L.W., Anger J.T., Gewertz B.L., Catchpole K. (2016). Barriers to efficiency in robotic surgery: The resident effect. J. Surg. Res..

[10] Wiegmann D.A., ElBardissi A.W., Dearani J.A., Daly R.C., Sundt Iii T.M. (2007). Disruptions in surgical flow and their relationship to surgical errors: An exploratory investigation. Surgery.

[11] Cofran L., Cohen T., Alfred M., Kanji F., Choi E., Savage S., Anger J., Catchpole K. (2021). Barriers to safety and efficiency in robotic surgery docking. Surg. Endosc..

[12] Neyens D.M., Bayramzadeh S., Catchpole K., Joseph A., Taaffe K., Jurewicz K., Khoshkenar A., San D., Group R.O.R.S. (2019). Using a systems approach to evaluate a circulating nurse’s work patterns and workflow disruptions. Appl. Ergon..

[13] Catchpole K., Neyens D.M., Abernathy J., Allison D., Joseph A., Reeves S.T. (2017). Framework for Direct Observation of Performance and Safety in Healthcare.

[14] Jain M., Cohen K., Shouhed D. (2020). Enhancing safety and efficiency in robotic surgery. Human Factors in Surgery.

[15] Bayramzadeh S., Joseph A., San D., Khoshkenar A., Taaffe K., Jafarifiroozabadi R., Neyens D.M., Group R.O.S. (2018). The impact of operating room layout on circulating nurse’s work patterns and flow disruptions: A behavioral mapping study. HERD Health Environ. Res. Des. J..

[16] Persson J., Dalholm E.H., Johansson G. (2014). Informing hospital change processes through visualization and simulation: A case study at a children’s emergency clinic. HERD Health Environ. Res. Des. J..

[17] Higuchi T.T., Gettman M.T. (2011). Robotic instrumentation, personnel and operating room setup. Atlas of Robotic Urologic Surgery.

